# Bakuchiol suppresses proliferation of skin cancer cells by directly targeting Hck, Blk, and p38 MAP kinase

**DOI:** 10.18632/oncotarget.7524

**Published:** 2016-02-20

**Authors:** Jong-Eun Kim, Jae Hwan Kim, Younghyun Lee, Hee Yang, Yong-Seok Heo, Ann M. Bode, Ki Won Lee, Zigang Dong

**Affiliations:** ^1^ Department of Agricultural Biotechnology and Research Institute for Agriculture and Life Sciences, Seoul National University, Seoul 151-742, Republic of Korea; ^2^ The Hormel Institute, University of Minnesota, MN 55912, USA; ^3^ Advanced Institutes of Convergence Technology, Seoul National University, Suwon 443-270, Republic of Korea; ^4^ Department of Chemistry, Konkuk University, Seoul 143-701, Republic of Korea

**Keywords:** bakuchiol, cell transformation, Blk, Hck, p38 MAPK

## Abstract

Bakuchiol is a meroterpene present in the medicinal plant *Psoralea corylifolia*, which has been traditionally used in China, India, Japan and Korea for the treatment of premature ejaculation, knee pain, alopecia spermatorrhea, enuresis, backache, pollakiuria, vitiligo, callus, and psoriasis. Here, we report the chemopreventive properties of bakuchiol, which acts by inhibiting epidermal growth factor (EGF)-induced neoplastic cell transformation. Bakuchiol also decreased viability and inhibited anchorage-independent growth of A431 human epithelial carcinoma cells. Bakuchiol reduced A431 xenograft tumor growth in an *in vivo* mouse model. Using kinase profiling, we identified Hck, Blk and p38 mitogen activated protein kinase (MAPK) as targets of bakuchiol, which directly bound to each kinase in an ATP-competitive manner. Bakuchiol also inhibited EGF-induced signaling pathways downstream of Hck, Blk and p38 MAPK, including the MEK/ERKs, p38 MAPK/MSK1 and AKT/p70^S6K^ pathways. This report is the first mechanistic study identifying molecular targets for the anticancer activity of bakuchiol and our findings indicate that bakuchiol exhibits potent anticancer activity by targeting Hck, Blk and p38 MAPK.

## INTRODUCTION

Skin cancer is the most commonly diagnosed cancer in the United States, and is categorized as either melanoma or nonmelanoma skin cancer (NMSC). Because NMSC is readily detectable at an early stage and has limited malignancy, its mortality is low compared to other cancers [1]. The most common types of NMSC are basal cell carcinoma (BCC) and squamous cell carcinoma (SCC). Changing environmental conditions, such as destruction of the ozone layer and environmental pollution, and alterations in eating habits with increased longevity have led to a rising incidence of NMSC [2], with approximately 1.3 million individuals currently suffering from the disease [3]. The annual cost of treating skin cancers in the U.S. is estimated at $4.8 billion for NMSC alone, with costs increasing rapidly in comparison to other cancers [4]. Thus, an improved chemopreventive agent is needed to prevent and treat NMSC and reduce healthcare costs [5].

The epidermal growth factor (EGF) receptor (EGFR) is an important mediator of skin cancer and belongs to the ErbB family of receptor tyrosine kinases (RTK) [6, 7]. Etiological factors for skin cancer such as ultraviolet light (UV), air pollution and toxic chemicals are known to activate this receptor. The EGFR tyrosine kinase domain activates downstream cellular signaling intermediates such as Src family kinases, the mitogen-activated protein (MAP) kinases and phosphoinositide 3-kinase (PI3-K). These signaling-induced cell transformation events are responsible for skin inflammation [6]. Previous studies have shown that EGFR is required for UV-induced NMSC development [8]. Both BCC and SCC highly overexpress EGFR, and inhibitors for EGFR downstream intermediates such as BRAF (vemurafenib) and Src (dasatinib) are used in clinical settings [9]. Kinases in the EGF-mediated signaling pathway are excellent targets for preventing skin cancer [10].

**Figure 1:**
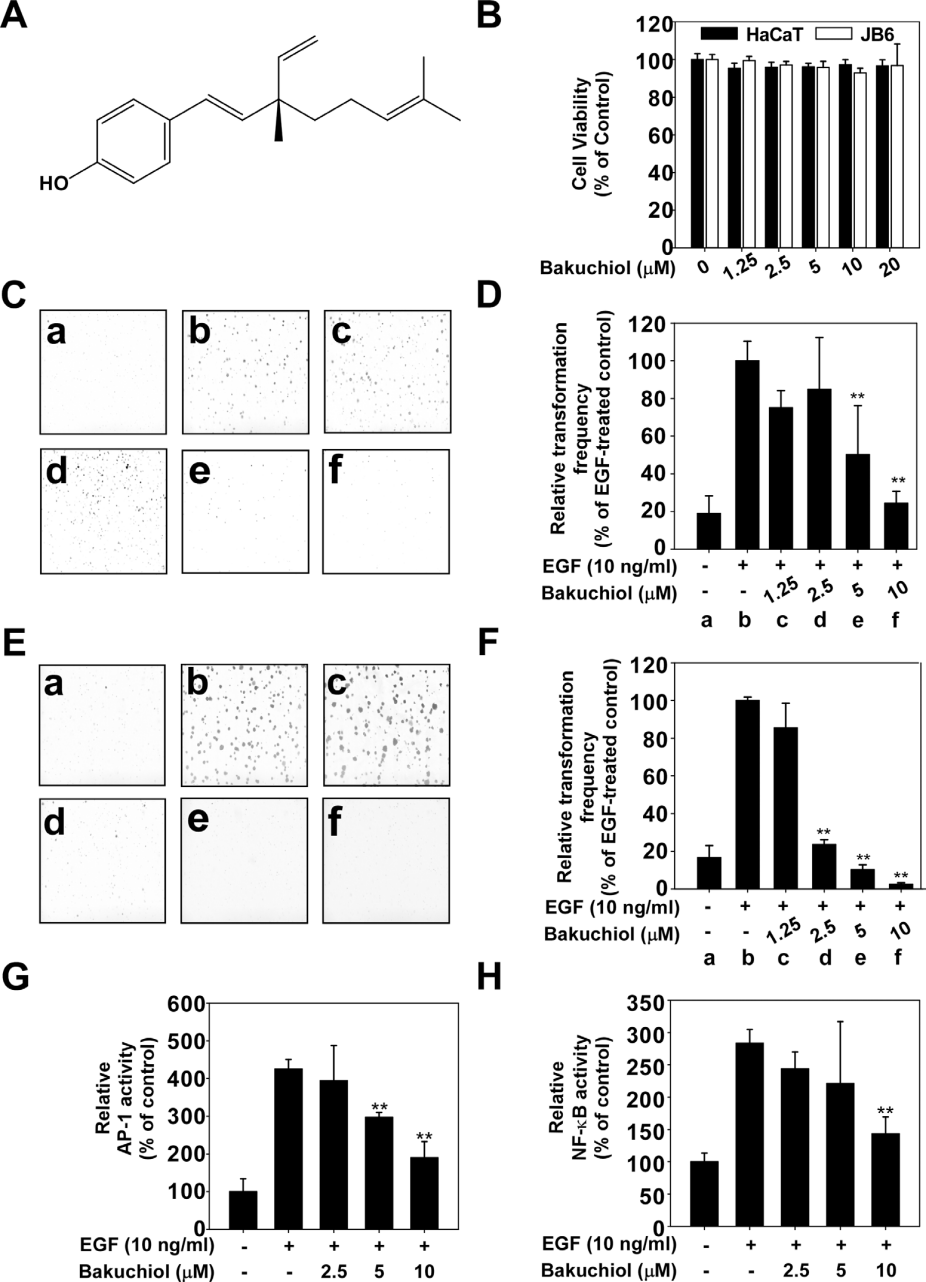
Bakuchiol inhibits epidermal growth factor (EGF)-induced neoplastic transformation of HaCaT and JB6 P+ cells (**A**) Chemical structure of bakuchiol. (**B**) Effect of bakuchiol on the viability of HaCaT and JB6 P+ cells. HaCaT and JB6 P+ cells were treated with bakuchiol (0, 1.25, 2.5, 5, 10 or 20 μM) for 72 h. Cell viability was measured by MTT assay as described in Materials and Methods. Bakuchiol inhibits EGF-induced neoplastic transformation of (**C**) and (**D**) HaCaT and (**E**) and (**F**) JB6 P+ cells. The effect of bakuchiol on EGF-induced cell transformation compared with untreated control cells (a); and cells treated with EGF alone (b); EGF and 1.25 μM Bakuchiol (c); EGF and 2.5 μM Bakuchiol (d); EGF and 5 μM Bakuchiol (e); or EGF and 10 μM Bakuchiol (f). The colonies were counted under a microscope with the aid of the Image-Pro Plus software program (vs. 6.2). Results are presented as mean values ± S.E. (*n* = 3). The asterisks (**) indicate a significant difference (*p* < 0.01) compared with the EGF-treated group. Bakuchiol suppresses EGF-induced (**G**) AP-1 and (**H**) NF-κB transactivation. JB6 P+ cells, which were stably transfected with *NF-κB or AP-1 luciferase reporter* plasmids, were pretreated with bakuchiol (0, 2.5, 5, or 10 μM) for 1 h before being exposed to EGF (10 ng/ml) and harvested 6 h later. Relative luciferase activities were determined and data are presented as mean values ± S.D. The asterisks (**) indicate a significant (**p < 0.01) inhibition of luciferase activity by bakuchiol compared to the group treated with EGF alone.

Bakuchiol (Figure 1A) is a meroterpene present in *Psoralea corylifolia*, which is a herb widely used in traditional Chinese and Ayurvedic medicine for curing premature ejaculation, knee pain, alopecia spermatorrhea, enuresis, backache, pollakiuria, vitiligo, callus, and psoriasis [11]. Of particular note, bakuchiol is also used as a cosmetic ingredient for antioxidant, anti-wrinkling, anti-acne and anti-fungal effects [12–14]. Bakuchiol also shows anticancer effects. Bakuchiol inhibits the proliferation of A549 human lung adenocarcinoma cells, SK-MEL-2 human melanoma cells and B16 mouse melanoma cells by inducing apoptosis [14–16]. A pharmacokinetics study showed that its C_max_ is approximately 1∼2 μM [17]. Therefore, bakuchiol could be a potential chemopreventive agent especially against skin cancer. However, the effect of bakuchiol on skin cancer and the underlying molecular mechanisms have not been fully investigated. In this study, we examined the effects of bakuchiol against skin cancer and provide solid evidence showing that Hck, Blk and p38 MAP kinases are novel targets of this compound to attenuate skin carcinogenesis.

## RESULTS

### Bakuchiol inhibits EGF-induced transformation of HaCaT and JB6 P+ cells

To elucidate the cancer chemopreventive effects of bakuchiol (Figure 1A), we used an EGF-induced cell transformation skin cell model comprised of a soft agar assay and human keratinocytes (HaCaT) and mouse epidermal JB6 P+ cells. Bakuchiol had no effect on the viability of these cells (Figure 1B) but inhibited EGF-induced cell transformation of HaCaT cells (5 μM; Figure 1C, 1D) and JB6 P+ cells (2.5 μM; Figure 1E, 1F). AP-1 and NF-κB are transcription factors that play crucial roles in EGF-induced cell transformation [18]. We measured AP-1 and NF-κB transactivation in JB6 P+ cells stably transfected with the *AP-1* or *NF-κB luciferase reporter* plasmid, respectively. Consistent with the above results for cell transformation, bakuchiol inhibited EGF-induced transactivation of AP-1 (Figure 1G) and NF-κB (Figure 1H)

### Bakuchiol attenuates EGF-induced signal transduction in HaCaT and JB6 P+ cells

**Figure 2:**
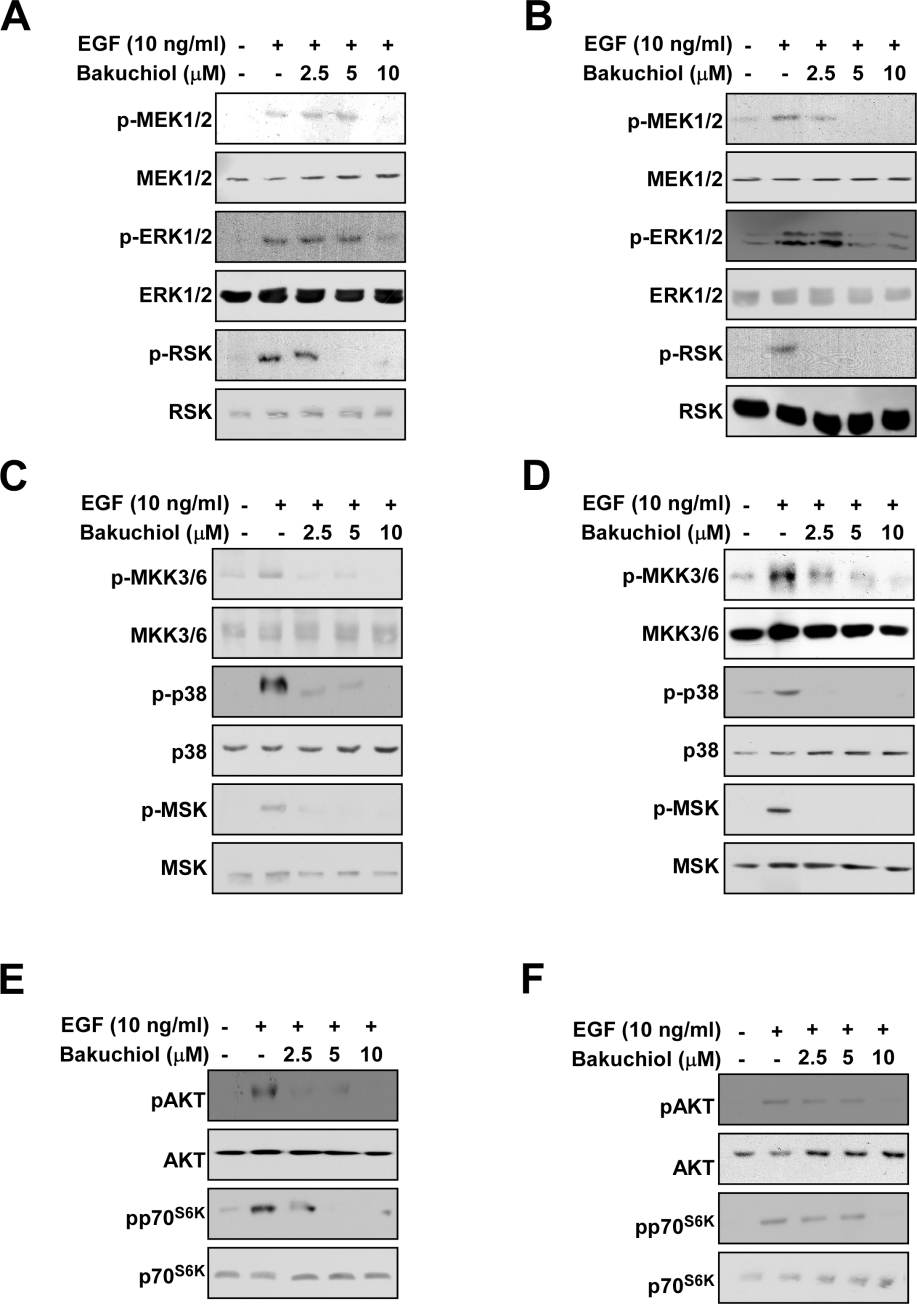
Effects of bakuchiol on EGF-induced signaling in HaCaT and JB6 P+ cells (**A**, **C**, and **E**) HaCaT and (**B**, **D**, and **F**) JB6 P+ cells were treated with bakuchiol (0, 2.5, 5, or 10 μM) for 1 h before treatment with EGF (10 ng/mL) and then harvested after 15 min. Immunoblot analysis was conducted as described in Materials and Methods. For each phosphorylated kinase, the total basal protein was used as a loading control.

Major signal transduction cascades that regulate EGF-induced AP-1 and NF-κB transactivation include the ERK1/2, p38 MAPK and AKT pathways [10, 19]. We measured the effects of bakuchiol on these pathways and found that bakuchiol inhibited EGF-induced ERK1/2 phosphorylation in HaCaT (Figure 2A) and JB6 P+ (Figure 2B) cells. Phosphorylation of MEK1/2 and p90^RSK^, upstream and downstream intermediates of ERK1/2, were also inhibited by bakuchiol in HaCaT (Figure 2A) and JB6 P+ (Figure 2B) cells. Another signaling pathway that regulates EGF-induced AP-1 and NF-κB transactivation is the p38 MAPK pathway. EGF-induced phosphorylation of MKK3/6-p38-MSK1 was inhibited by bakuchiol in HaCaT (Figure 2C) and JB6 P+ (Figure 2D) cells. Bakuchiol also inhibited EGF-induced AKT and p70^S6K^ phosphorylation in HaCaT (Figure 2E) and JB6 P+ (Figure 2F) cells. These results suggest that the inhibition of these pathways by bakuchiol leads to the suppression of AP-1 and NF-κB activities, resulting in decreased neoplastic transformation.

### Hck, Blk and p38 MAPK are direct molecular targets of bakuchiol

**Figure 3:**
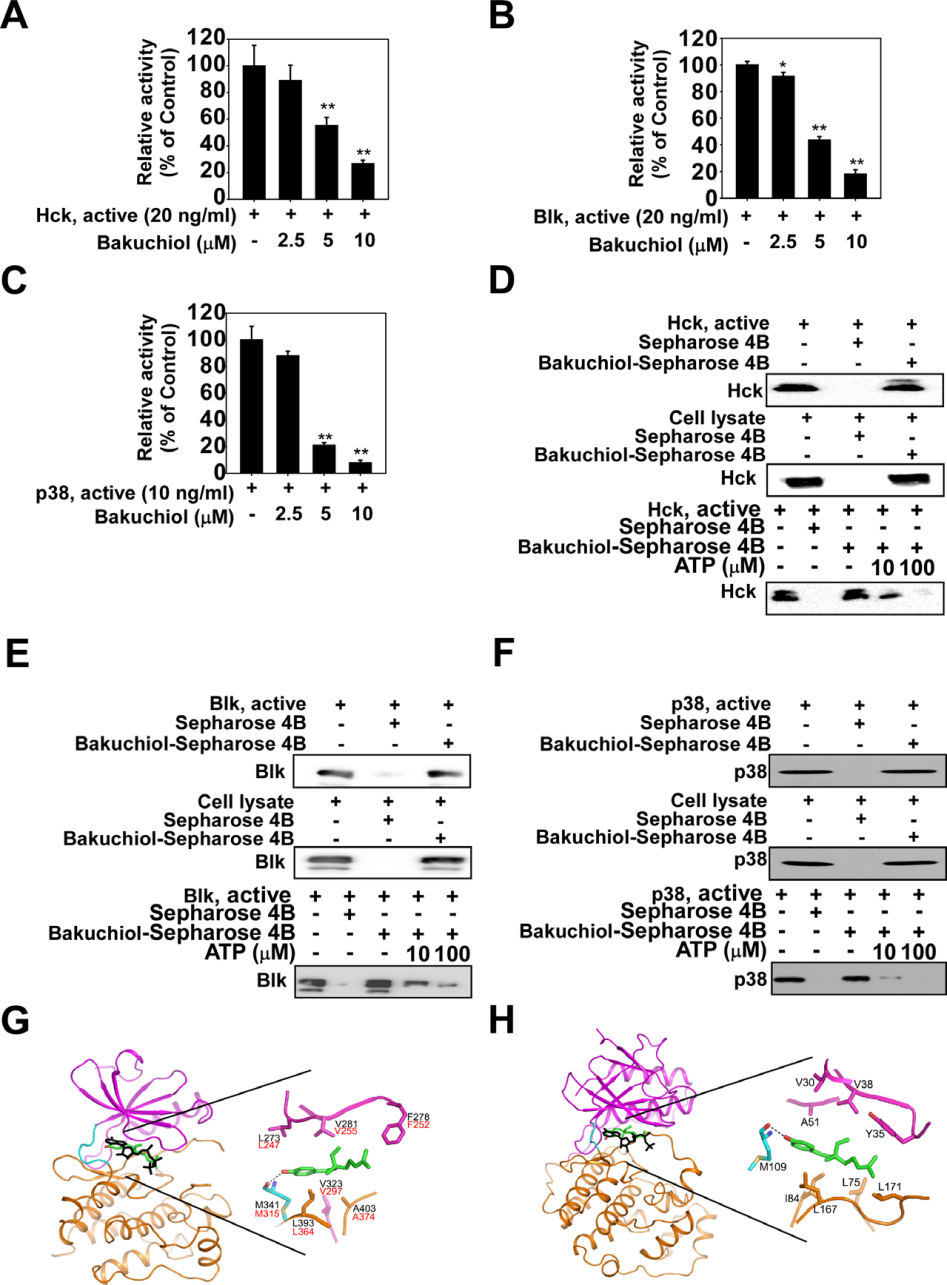
Bakuchiol inhibits kinase activity of Hck, Blk and p38 mitogen activated protein kinase (MAPK) by competing with ATP for binding Bakuchiol inhibits (**A**) Hck, (**B**) Blk and (**C**) p38 MAPK activity. Bakuchiol binds (**D**) Hck, (**E**) Blk or (**F**) p38 MAPK directly in an ATP-competitive manner. Active Hck (20 ng), Blk (20 ng) or p38 MAPK (10 ng) or respective cell lysates were mixed with Sepharose 4B only or Sepharose 4B conjugated with bakuchiol (0, 2.5, 5, or 10 μM) and then incubated with or without [γ^−32^P] ATP. For ATP measurement, the radioactive incorporation was determined using a scintillation counter. Hck, Blk and p38 MAPK and bakuchiol binding was confirmed by immunoblotting with an antibody against (D) Hck, (E) Blk and (F) p38 MAPK (upper panels): lane 1 (input control), Hck, Blk and p38 MAPK protein standard; lane 2 (negative control), Sepharose 4B beads only were used for an immunoprecipitation assay; lane 3, Hck, Blk or p38 MAPK was immunoprecipitated using bakuchiol-Sepharose 4B beads. Bakuchiol binds Hck, Blk and p38 MAPK in cell lysates (middle panels). Binding of bakuchiol to Hck, Blk and p38 MAPK in HaCaT cells was confirmed by immunoblotting with appropriate antibodies. Lane 1 (input control), HaCaT cell lysate; lane 2 (negative control), HaCaT cell lysates were precipitated with Sepharose 4B beads; lane 3, HaCaT cell lysates were precipitated using bakuchiol-Sepharose 4B beads. Active Hck, Blk or p38 MAPK (0.2 μg; lower panels) was incubated with ATP at the indicated concentrations (0, 10, or 100 μM) together with 100 μl bakuchiol-Sepharose 4B beads or Sepharose 4B beads (negative control) added in reaction buffer to a final volume of 500 μl. The immunoprecipitated proteins were detected by immunoblotting with antibodies against Hck, Blk or p38 MAPK. Lane 1, negative control, showing that Hck, Blk or p38 MAPK does not bind to Sepharose 4B beads alone; lane 2: positive control, showing that Hck, Blk or p38 MAPK binds with bakuchiol-Sepharose 4B beads. (**G**, **H**) Hypothetical computer-generated models of Hck, Blk or p38MAPK in complex with bakuchiol. (G) Model structure of the kinase domain of Hck in complex with bakuchiol and an enlarged view. Bakuchiol (atomic color) binds to the ATP binding site, and ATP (black) is overlaid for comparison. The residues of Hck involved in the interaction with bakuchiol are labeled in black and the corresponding residues of Blk are labelled in red (G). (H) Model structure of p38 MAPK in complex with bakuchiol and an enlarged view. Bakuchiol (atomic color) binds to the ATP binding site of p38 and ATP (black) is overlaid for comparison. The N-lobe, C-lobe, and hinge loop of the protein kinases are colored violet, orange, and cyan, respectively. The hydrogen bonds are depicted as dotted lines (G, H).

To identify the molecular targets of bakuchiol, we screened 78 cancer-related kinases using KinaseProfiler provided by EMD Millipore. Results of the screening with 20 μM bakuchiol indicated that Hck, Blk and p38 MAPK are inhibited by over 40% (Table 1), with activity reduced in a concentration-dependent manner (Figure 3A, 3B, 3C). To identify the mechanism by which bakuchiol modulates Hck, Blk and p38 MAPK kinase activities, we examined whether bakuchiol binds directly to these targets. Pull-down assay results revealed that bakuchiol physically binds to the active Hck, Blk or p38 MAPK (Figure 3D, 3E, 3F, upper panels, lane 3), but not to unconjugated Sepharose 4B beads (Figure 3D, 3E, 3F, upper panels, lane 2). The input lane (Figure 3D, 3E, 3F, upper panels, lane 1) showing the loading of 20 ng of the active protein as a marker, suggested that the detected band was indeed the indicated protein. We also observed binding of bakuchiol to Hck, Blk and p38 MAPK in HaCaT cells (Figure 3D, 3E, 3F, middle panels). Next, to examine the mode of bakuchiol binding to Hck, Blk and p38 MAPK, we performed ATP competitive-binding assays. ATP competed with bakuchiol for Hck, Blk and p38 MAPK binding (Figure 3D, 3E, 3F, bottom panels), indicating that bakuchiol binds to or otherwise interferes with the respective Hck, Blk and p38 MAPK ATP-binding pocket. Based on the experimental finding that bakuchiol binds to Hck, Blk and p38 MAPK in an ATP-competitive manner, we conducted computer modeling studies to investigate the binding modes of bakuchiol with these proteins using the crystal structures of Hck and p38α MAPK as described in Materials and Methods. Hck and Blk have a conserved binding region with bakuchiol and thus we performed computer modeling studies for Hck and p38α MAPK (Figure 3G, 3H).

**Table 1:** Kinase profiling of Bakuchiol (20 μM)

Kinase	Activity	Kinase	Activity	Kinase	Activity	Kinase	Activity	Kinase	Activity	Kinase	Activity
Abl	133	CDK9/cyclin T1	94	EGFR (T790M)	119	Lck	71	MST1	62	Rsk2	93
ASK1	68	CHK1	136	EGFR (T790M, L858R)	107	LKB1	85	mTOR	108	p38	51
Aurora-A	66	CHK2	97	FAK	100	Lyn	134	NEK7	102	SGK	151
Blk	47	CHK2 (I157T)	91	Fgr	98	MAPK1	78	p70S6K	172	SIK	108
CaMKI	85	CHK2 (R145W)	110	Flt1	93	MAPK2	113	PDK1	75	Src	95
CaMKIIβ	65	cKit	92	Fyn	102	MEK1	87	Pim-1	111	Syk	67
CDK1/cyclin B	84	CSK	147	GSK3β	49	Met	70	Pim-2	122	WNK2	82
CDK2/cyclin A	90	c-RAF	101	Hck	57	MKK4	108	Pim-3	80	Yes	134
CDK2/cyclin E	76	cSRC	87	IKKα	148	MKK6	94	PKA	120	ZAP-70	102
CDK3/cyclin E	100	DAPK1	80	JAK2	100	MKK7β	94	PKBα	109		
CDK5/p25	94	DDR2	97	JAK3	79	MLK1	59	PKCα	109		
CDK5/p35	111	EGFR	111	JNK1α1	98	Mnk2	84	Plk1	88		
CDK6/cyclin D3	108	EGFR (L858R)	101	JNK2α2	100	MSK1	118	ROCK-I	84		
CDK7/cyclin H	105	EGFR (L861Q)	105	KDR	92	MSK2	101	Rsk1	80		

### Bakuchiol decreases viability and suppresses anchorage-independent growth of A431 cells

**Figure 4:**
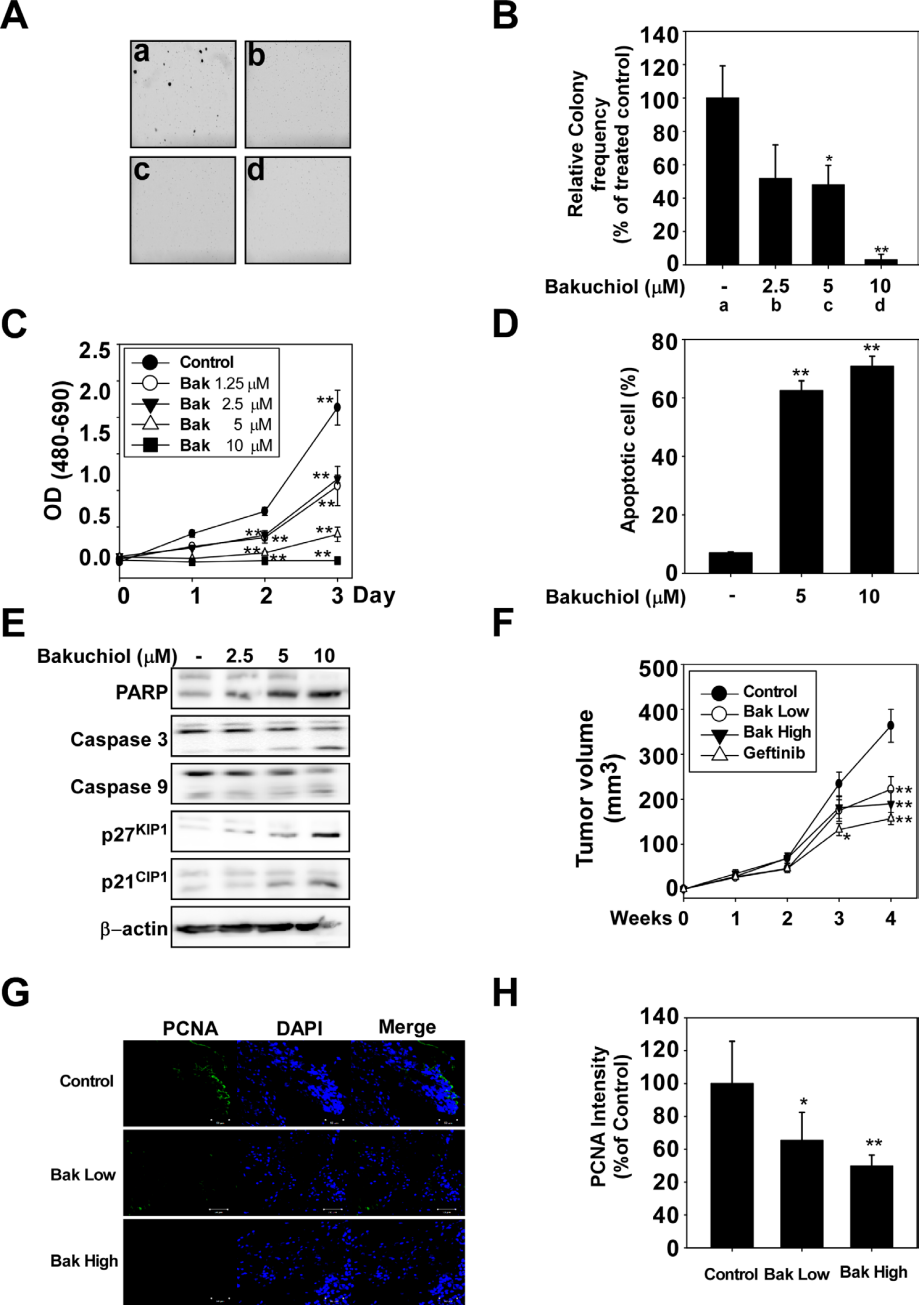
Bakuchiol suppresses growth of A431 xenograft tumors in nude mice Bakuchiol decreases viability and inhibits anchorage-independent A431 cell growth. (**A**, **B**) Bakuchiol inhibits anchorage-independent growth of A431 cells. A soft agar assay was performed with bakuchiol treatment (0, 2.5, 5, or 10 μM) and the number of colonies was counted under a microscope with the aid of the Image-Pro Plus software program (v. 6.2). Results are represented as mean values ± S.E. (*n* = 3). The asterisks (*, **) indicate a significant difference (*p* < 0.05, *p* < 0.01) compared with the untreated control group. (**C**) Bakuchiol decreases viability of A431 cells. The MTT assay was used to evaluate viability of cells treated with bakuchiol (0, 2.5, 5, or 10 μM). (**D**) Bakuchiol induces apoptosis of A431 cells. Apoptosis was analyzed by Annexin V/PI staining. Bakuchiol induces apoptosis in A431 cells in a dose-dependent manner. For C and D, the asterisks (**) indicate a significant difference (*p* < 0.01) compared with the untreated control group. (**E**) Bakuchiol stimulates apoptosis-related signaling pathways. A431 cells were treated with bakuchiol (0, 2.5, 5, or 10 μM) for 48 h and harvested. Immunoblotting was conducted using specific antibodies. (**F**) The average tumor volume of control and bakuchiol-treated mice plotted over 28 days after tumor cell inoculation. The *p* values indicate statistical significance for the inhibition of tumor growth by bakuchiol (**p < 0.01). (**G**, **H**) Representative images and quantification of PCNA (green) and DAPI (blue) staining of A431 xenograft tumors (*n* = 12). Scale bars = 100 μm. Data are represented as mean values ± S.E. The asterisks (*, **) indicate a significant (*p* < 0.05, *p* < 0.01) difference between the vehicle-treated and bakuchiol-treated groups.

To confirm the effect of bakuchiol in an animal model, we used A431 skin epidermoid carcinoma cells. Because the A431 cell line highly overexpresses EGFR, forms colonies when cultivated in soft agar, and develops tumors in nude mice, it serves as an excellent model for studying EGFR-mediated cellular signaling [10]. Bakuchiol inhibited anchorage-independent (Figure 4A, 4B) and decreased viability (Figure 4C) of A431 cells, as well as signal transduction in these cells in a similar pattern to that observed for HaCaT and JB6 P+ cells (Supplementary Figure 1). Next, we measured the effect of bakuchiol on apoptosis and found that bakuchiol induced apoptosis of A431 cells (Figure 4D) and activated apoptosis-associated proteins, including PARP, caspase 3, caspase 9, p27^KIP1^ and p21^CIP1^ (Figure 4E).

### Bakuchiol suppresses growth of A431 xenograft tumors in nude mice

We examined the effects of bakuchiol in an *in vivo* xenograft mouse model. The average volume of tumors in vehicle-injected mice reached 380 mm^3^ at 4 weeks post-injection. In contrast, the average tumor volume was only 221 or 198 mm^3^ in mice treated with 10 or 40 mg/kg bakuchiol, respectively (Figure 4F). Immunostaining demonstrated that levels of PCNA, a cell proliferation marker, were lower in the bakuchiol-treated A431 tumor groups compared to the vehicle-treated groups (Figure 4G, 4H).

### Hck and Blk knockdown inhibits EGF-induced cell transformation in HaCaT and JB6 P+ cells and anchorage-independent and -dependent cell growth of A431 cells

**Figure 5:**
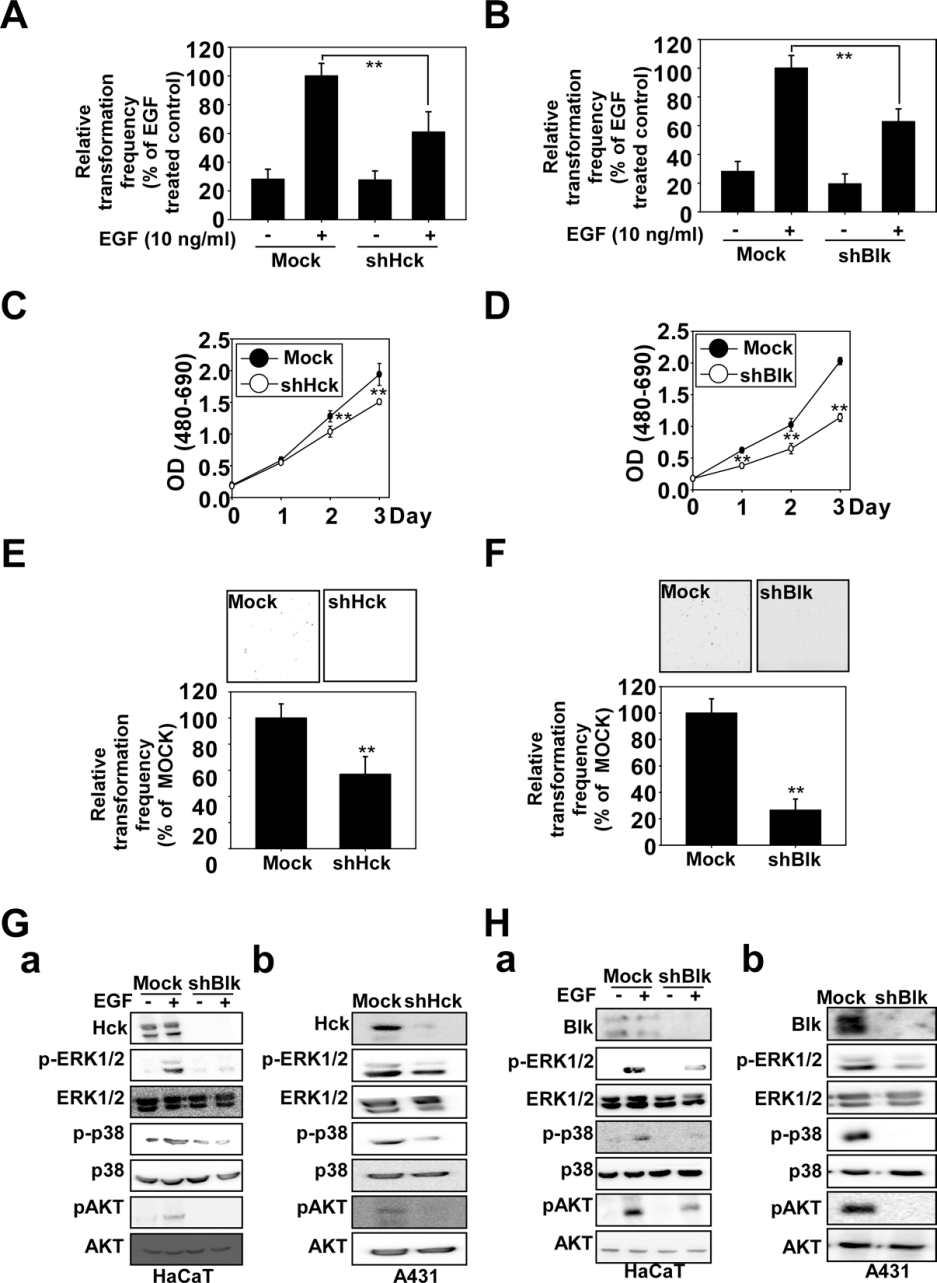
Knockdown of Hck or Blk inhibits EGF-induced neoplastic growth of HaCaT cells and growth of A431 cells A soft agar assay was performed using mock or (**A**) shBlk- or (**B**) shHck-transfected HaCaT cells and the number of colonies was counted under a microscope with the aid of the Image-Pro Plus software program (vs. 6.2). Knockdown of Blk or Hck decreases viability and inhibits anchorage-independent cell growth of A431 cells. Viability was evaluated by MTT assay using mock- or (**C**) shHck- or (**D**) shBlk- transfected A431 cells. A soft agar assay was performed using mock or (**E**) shHck - or (**F**) shBlk -transfected A431 cells. The asterisks (**) indicate a significant (**p < 0.01) difference between mock- and shHck- or shBlk-transfected cells. Knockdown of (**G**) Hck or (**H**) Blk regulates the Hck and Blk downstream signaling cascades. Knockdown of Hck or Blk inhibits ERK1/2, p38 and AKT phosphorylation in EGF-treated (a) HaCaT cells or (b) A431 cells. The cells were seeded and incubated for 48 h before proteins were recovered. Mock-, shHck- or shBlk-transfected HaCaT cells were treated with EGF (10 ng/ml) and harvested after 15 min. Immunoblotting was conducted using specific antibodies.

The role of p38 MAPK in EGF-induced cell transformation and skin cancer growth has been previously described [20, 21]. In this study, we observed that Hck and Blk are important targets for chemoprevention. Using lentiviral infection, we established HaCaT and A431 cells stably expressing shMock, shHck or shBlk. EGF-induced cell transformation was decreased in shHck - (Figure 5A and Supplementary Figure 2A) and shBlk - (Figure 5B and Supplementary Figure 2B) expressing HaCaT cells. In addition, the effect of shHck knockdown (Figure 5C, 5E) or shBlk (Figure 5D, 5F) expression on anchorage-independent and viability of A431 cells was assessed. These results showed that anchorage-independent growth was strongly inhibited and viability was decreased by knocking down the expression of shHck or shBlk. We next investigated the effects of knocking down Hck or Blk on EGF-induced signal transduction in HaCaT cells and signal transduction in A431 cells. Notably, compared with HaCaT and A431 cells expressing green fluorescent protein–shRNA (shMock), HaCaT and A431 cells expressing shHck or shBlk exhibited a substantially reduced abundance of endogenous Hck (Figure 5G) or Blk (Figure 5H). Knockdown of Hck or Blk reduced EGF-induced ERK1/2, p38, or AKT phosphorylation (Figure 5GA, 5HA) in HaCaT cells, as well as inhibited the phosphorylation of ERK1/2, p38, and AKT in A431 cells (Figure 5GB, 5HB).

## DISCUSSION

Over the past two decades, single-target drugs have become a major paradigm of drug development because selective ligands can help to avoid unwanted side effects [22]. After the success of the prescription drug Gleevec, pharmaceutical researchers have paid significant attention to the potential of multiple-target inhibitors for a variety of applications [23]. Complex diseases such as cancer are not likely to result from a single defect, owing to multiple pathogenic mechanisms being responsible [24]. However, as the number of inhibitory targets increases, side effects for these therapies can also increase. In this study, we observed that bakuchiol is a multi-target kinase inhibitor that targets Hck, Blk and p38 MAPK activities by direct binding. Bakuchiol has been consumed as a natural foodstuff for centuries and is regarded as safe [11]. Therefore, bakuchiol could be an excellent chemopreventive agent for skin cancer.

In this study, using a kinase array, we found that Hck, Blk and p38 MAPK are direct targets of bakuchiol. The p38 MAPK is one of the MAPKs that plays an important role in cellular responses to external stress signals, such as proliferation, differentiation, death, migration and invasion. Our previous studies revealed that p38 MAPK plays an important role in EGF-induced cell transformation and skin cancer development by activating the AP-1 transcription factor [20, 21]. The p38 MAPK is activated by cellular stresses including growth factors, UV, and inflammatory cytokines, while p38 MAPK blockade has been shown to yield fewer and smaller tumors in models of solar UV-induced skin carcinogenesis [25]. Therefore, inhibition of p38 MAPK represents a promising strategy for attenuating skin cancer. The roles of Hck and Blk in cancers have been investigated [26–32]. Knocking down Hck substantially enhanced killing of cancer cells by the drug, SS1P, which is a recombinant immunotoxin (RIT) that targets mesothelin in mesothelin-transfected A431 cells [30]. The Hck tyrosine kinase regulates toll-like receptor 4-induced tumor necrosis factor and interleukin-6 production mediated by AP-1 in primary peripheral blood mononuclear cells [33]. Blk is expressed ectopically in patients with early and late-stage cutaneous T-cell lymphoma (CTCL) and promotes the proliferation of malignant CTCL cells [34]. However, the roles of Hck and Blk in skin cancer have not yet been elucidated. In this study, we examined the roles of Hck and Blk in EGF-induced skin carcinogenesis. Knockdown of Hck or Blk inhibited EGF-induced transformation of HaCaT cells and anchorage-independent growth of A431 cells. Knockdown of Hck or Blk inhibited ERK1/2 and AKT phosphorylation in EGF-treated HaCaT and A431 cells, which indicates that Hck and Blk act as upstream mediators of the MAPK and PI3-K pathways. The cellular signaling in EGF-treated HaCaT and A431 cells was similar between bakuchiol treatment and silenced Hck or Blk. Therefore, Hck and Blk could be potential targets to prevent skin carcinogenesis and indicate that Hck, Blk and p38 MAPK are targets of bakuchiol in skin carcinogenesis.

To elucidate the mechanism as to how bakuchiol inhibits Hck, Blk and p38 MAPK activities, we conducted a computational modeling study. In the modeling structure of the kinase domain of Hck in complex with bakuchiol, the hydroxyl group at the benzene ring of the compound formed a hydrogen bond with the backbone carbonyl groups of Met341 in the hinge loop (Figure 4G). In addition, bakuchiol could theoretically make hydrophobic interactions with the side chains of Leu273, Phe278, Val281, and Val323 from the N-lobe and Leu393 and Ala403 from the C-lobe. No crystal structure of the kinase domain of Blk was available for the modeling study. However, its sequence homology with Hck is very high (75% identity) and all the residues that are thought to interact with bakuchiol are conserved between Hck and Blk, implying that the binding mode of bakuchiol in Hck and Blk could be almost identical. In the modeling structure of p38α in complex with bakuchiol, the hydroxyl group at the benzene ring of the compound formed a hydrogen bond with the backbone carbonyl groups of Met109 in the hinge loop (Figure 4H). In addition, the inhibitor would be sandwiched by the side chains of the hydrophobic residues in the ATP-binding site, including Val30, Tyr35, Val38, and Ala51 from the N-lobe and Leu75, Ile84, Leu167, and Leu171 from the C-lobe. The strong inhibitory activity of bakuchiol against Hck, Blk and p38α is due to these hydrogen bonds and hydrophobic interactions. Further studies with X-ray crystallography to determine the complex structure with bakuchiol would elucidate its exact binding mode with Hck, Blk and p38α. In summary, bakuchiol inhibits EGF-induced neoplastic transformation of HaCaT and JB6 P+ cells and also reduces tumor growth in an A431 mouse xenograft model. This inhibition is mediated through the blocking of the MEK/ERK/p90RSK, MKK3/6-p38-MSK1 and PI3-K/AKT/p70^S6K^ signaling pathways and the subsequent suppression of AP-1 activity. Bakuchiol also strongly suppressed Hck, Blk, and p38 MAPK activities. Overall, these results suggest that Hck, Blk and p38 MAPK are important molecular targets for the suppression of neoplastic transformation by bakuchiol.

## MATERIALS AND METHODS

### Materials

Bakuchiol (98%) was purchased from LKT Laboratories (St. Paul, MN) and EGF was obtained from Calbiochem (Darmstadt, Germany). Eagle's minimum essential medium (MEM), gentamicin, and L-glutamine were obtained from Gibco–BRL (Carlsbad, CA). Fetal bovine serum (FBS) was purchased from Gemini Bio-Products (Calabasas, CA). Antibodies against phosphorylated p90RSK (Thr359/Ser363), phosphorylated AKT (Ser473), total AKT, phosphorylated p70^S6K^, total p70^S6K^, and total p90^RSK^ were purchased from Cell Signaling Technology, Inc. (Beverly, MA). Antibodies against phosphorylated ERK1/2 (Thr202/Tyr204) and total ERKs were obtained from Santa Cruz Biotechnology (Santa Cruz, CA). The antibody against β-actin was from Sigma-Aldrich (St. Louis, MO) and the active Hck, Blk and p38 MAPK proteins were obtained from EMD Millipore (Billerica, MA). 3-[4, 5-dimethylatiazol-2-yl]-2,5 diphenyltetrazolium bromide (MTT) powder was purchased from USB Co. (Cleveland, OH). ATP and the chemiluminescence detection kit were purchased from GE Healthcare Biosciences (Pittsburgh, PA) and the protein assay kit was obtained from Bio-Rad Laboratories (Hercules, CA).

### Cell culture

JB6 P+ mouse skin epidermal cells (5% FBS-MEM), HaCaT cells and A431 cells were cultured at 37°C and 5% CO_2_ in growth medium (10% FBS-DMEM) supplemented with antibiotics. Cells were maintained by subculturing at 80 to 90% confluence and media were changed every 3 days.

### Anchorage-independent cell transformation assay

A431 cells were suspended in Basal Minimal Eagle (BME) medium and added to 0.6% agar, with the indicated concentrations of bakuchiol in the base layer and in a top layer of 0.3% agar. JB6 P+ and HaCaT cells were further exposed to epidermal growth factor (EGF; 10 ng/ml) together with bakuchiol treatment or vehicle control. The cultures were maintained at 37°C in a 5% CO_2_ incubator for 1–2 weeks and then colonies were counted under a microscope using Image-Pro Plus software (V.4) (Media Cybernetics, Silver Spring, MD).

### Cell viability

Cells were seeded in 96-well plates, incubated for 24 h and then treated with the indicated doses of each compound. After incubation, cell viability was measured by MTT assay or CellTiter96 AQueous One Solution (Promega, Madison, WI).

### Western blot assays

Cells (1.5 × 10^6^) were cultured in 100-mm dishes for 48 h, and then serum starved in 0.1% FBS-MEM for 24 h. The cells were then treated with bakuchiol (0, 5, 10, and 20 μM) for 1 h before exposure to 10 ng/ml EGF for an additional 30 min. Cells were harvested and disrupted with lysis buffer before protein concentration was measured using a dye-binding protein assay kit (Bio-Rad Laboratories) as described in the manufacturer's manual. Protein lysate (40 μg) was subjected to 10% sodium dodecyl sulfate–polyacrylamide gel electrophoresis (SDS-PAGE) and transferred to a polyvinylidene difluoride membrane (PVDF; EMD Millipore). After transfer, the membranes were incubated with specific primary antibodies at 4°C overnight. Protein bands were visualized by a chemiluminescence detection kit after hybridization with a horseradish peroxidase (HRP)-conjugated secondary antibody.

### Kinase assay

The *in vitro* kinase assay was conducted in accordance with the instructions provided by Millipore. Active kinases were mixed with bakuchiol (0, 5, 10, 20 μM) in reaction buffer [40 mM MOPS/NaOH (pH 7.0), 1 mM EDTA, 10 mM MnCl_2_, and 0.8 M L-ammonium sulphate]. The mixture was incubated with 100 μM substrate for 5 min at room temperature followed by incubation with 10 μL of a ATP mixture (25 mM MgAc and 0.25 mM ATP-containing 10 μCi [γ^−32^P] ATP) for 20 min at 30°C and then 25 μl of reaction mixture were transferred onto P81 filter papers (EMD Millipore). The filter papers were washed twice with 0.75% phosphoric acid and once with acetone. The radioactive incorporation was determined using a scintillation counter.

### *In vitro* pull down assays

For the preparation of bakuchiol–Sepharose 4B beads, Sepharose 4B powder was suspended in 1 mM HCl and bakuchiol was added to the coupling solution (0.1 M NaHCO_3_ and 0.5 M NaCl) and mixed on a rotary shaker at 4°C overnight. The procedure was performed as described previously [35]. For the *in vitro* and *ex vivo* pull down assays, Hck, Blk or p38 MAPK protein and JB6 P+ cell lysates were incubated with bakuchiol–Sepharose 4B (or Sepharose 4B alone as a control) beads in reaction buffer and mixed on a rotary shaker at 4°C overnight. After incubation, the beads were washed 5 times with washing buffer. Proteins bound to the beads were analyzed by Western blotting.

### Lentiviral infection

The lentiviral expression vectors and packaging vectors, including *pMD2.0G* and *psPAX*, were purchased from Addgene Inc. (Cambridge, MA). To prepare viral particles, each viral vector and packaging vector (*pMD2.0G* and *psPAX*) were transfected into 293T cells using JetPEI following the manufacturer's suggested protocols. The transfection medium was changed at 24 h after transfection and cells were cultured for 36 h. The viral particles were harvested by filtration using a 0.45 mm syringe filter, then combined with 8 μg/ml of polybrane (EMD Millipore) and infected into 60% confluent HaCaT and A431 cells overnight. The cell culture medium was replaced with fresh complete growth medium for 24 h and the cells were selected with puromycin (1.5 μg/ml) for 48 h. The selected cells were used for experiments after confirming expression by Western blot.

### Computational modeling

The crystal coordinates of Hck (PDB entry 1AD5) and p38α (PDB entry 1OUY) were used for the simulated docking of bakuchiol. Insight II (Accelrys Inc, San Diego, CA) was used for the modeling study and structure analysis.

### Xenograft mouse model

The experimental protocol was approved by the Animal Care and Use Committee of Seoul National University (SNU-140711-4). Male and female mice (6-week-old, BALB/c-nu) implanted with A431 cells were used for the xenograft assay. A431 (1 × 10^6^) cells, suspended in 100 uL of serum-free media containing 50% Matrigel (BD Biosciences), were implanted subcutaneously in both dorsal flanks of the mice. Cells were allowed to form tumors, and once the tumors reached a size of 50 mm^3^, the mice were randomly assigned into groups (8 mice/group). Bakuchiol or gefitinib suspended in vehicle (10% DMSO in PBS) was administered at a dose of 5 (low), 10 (high) or 5 (gefitinib) mg/kg intraperitoneally 5 days a week. Tumor volumes were measured every week using calipers and calculated according to a standard formula: V = (L × H × W)π/6. Tumor tissues were preserved for further analysis

### Immunostaining analysis

The immunostaining assay was conducted as described previously.^26^ Briefly, tumor samples were frozen using tissue-freezing medium immediately after dissection and stored at −70°C. Cryostat sections were cut into fragments of 10 μm thickness and fixed with cold acetone for 10 min at RT and left to dry. Specimens were incubated in 5% goat serum in PBS containing 0.3% Triton X-100 (PBS-T) for 1 h at room temperature for permeabilization and to block any non-specific antibody binding. Primary antibodies were incubated at 4°C overnight, and secondary antibodies for 2 h at room temperature. Fluorescent images were obtained by confocal microscopy and signal-positive area densities were measured by analysis of pixel-based fluorescence intensities using the ImageJ software program (NIH).

### Statistical analysis

As necessary, data are expressed as means ± S.E.M. or S.D. and significant differences were determined using one-way ANOVA (Analysis of Variance). Duncan's multiple range test was used to determine which mean values were significantly different. A probability value of *p* < 0.05 was used as the criterion for statistical significance.

## SUPPLEMENTARY MATERIALS FIGURES


