# Supplementary material for: Bakuchiol suppresses proliferation of skin cancer cells by directly targeting Hck, Blk, and p38 MAP kinase
Source: Oncotarget. 2016 Feb 20;7(12):14616–27. doi: 10.18632/oncotarget.7524 (PMC4924739; doi:10.18632/oncotarget.7524)
Supplement: Supplementary file 1 [file oncotarget-07-14616-s001.pdf]

Bakuchiol suppresses proliferation of skin cancer cells by directly targeting Hck, Blk, and p38 MAP kinase

Supplementary Materials

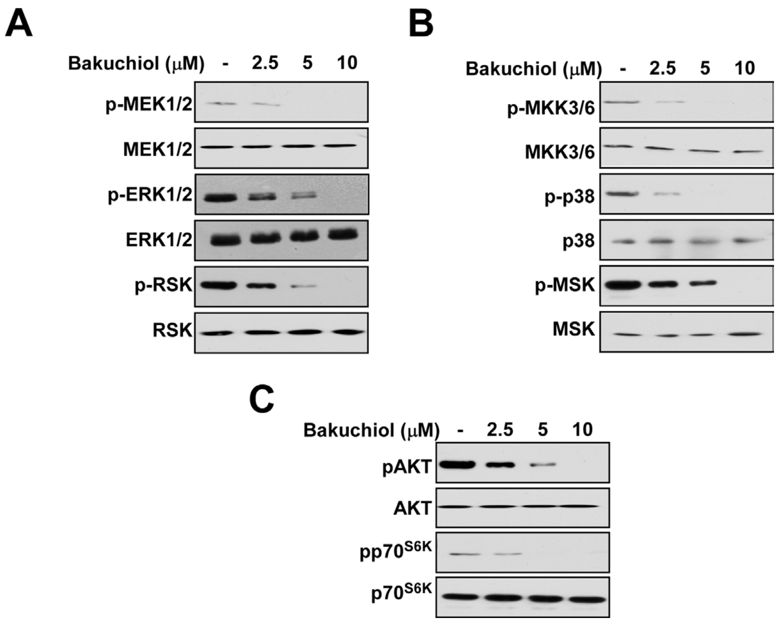

Supplementary Figure S1: Effects of bakuchiol on signal transduction in A431 cells. A431 cells were treated with bakuchiol (0, 2.5, 5, or 10 μM) for 48 h and harvested. Immunoblotting was conducted using specific antibodies.

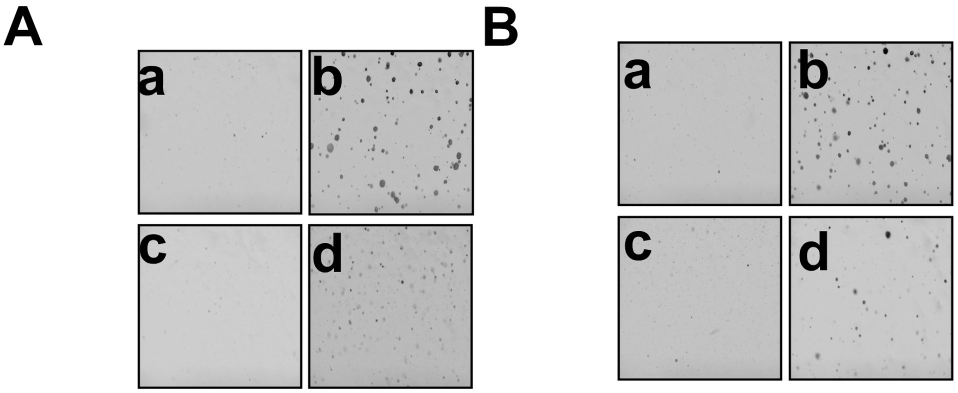

Supplementary Figure S2: Corresponds to quantitative data shown in Figure 6A and 6B.
